# Voice disorder in systemic lupus erythematosus

**DOI:** 10.1371/journal.pone.0175893

**Published:** 2017-04-17

**Authors:** Milena S. F. C. de Macedo, Kauê M. Costa, Manoel da Silva Filho

**Affiliations:** 1 Institute of Biological Sciences, Federal University of Pará, Belém, Pará, Brazil; 2 Ophir Loyola Hospital, Belém, Pará, Brazil; 3 Institute for Neurophysiology, Interdisciplinary Center for Neuroscience, Goethe University, Frankfurt am Main, Germany; Peking University First Hospital, CHINA

## Abstract

Systemic lupus erythematosus (SLE) is a chronic disease characterized by progressive tissue damage. In recent decades, novel treatments have greatly extended the life span of SLE patients. This creates a high demand for identifying the overarching symptoms associated with SLE and developing therapies that improve their life quality under chronic care. We hypothesized that SLE patients would present dysphonic symptoms. Given that voice disorders can reduce life quality, identifying a potential SLE-related dysphonia could be relevant for the appraisal and management of this disease. We measured objective vocal parameters and perceived vocal quality with the GRBAS (Grade, Roughness, Breathiness, Asthenia, Strain) scale in SLE patients and compared them to matched healthy controls. SLE patients also filled a questionnaire reporting perceived vocal deficits. SLE patients had significantly lower vocal intensity and harmonics to noise ratio, as well as increased jitter and shimmer. All subjective parameters of the GRBAS scale were significantly abnormal in SLE patients. Additionally, the vast majority of SLE patients (29/36) reported at least one perceived vocal deficit, with the most prevalent deficits being vocal fatigue (19/36) and hoarseness (17/36). Self-reported voice deficits were highly correlated with altered GRBAS scores. Additionally, tissue damage scores in different organ systems correlated with dysphonic symptoms, suggesting that some features of SLE-related dysphonia are due to tissue damage. Our results show that a large fraction of SLE patients suffers from perceivable dysphonia and may benefit from voice therapy in order to improve quality of life.

## Introduction

As medical research progresses many incurable diseases with a previously high degree of mortality become manageable with chronic care. A major challenge therein is the development of therapeutic schedules that preserve patient wellbeing over the extended life spans. Systemic lupus erythematosus (SLE), a chronic disease in which noxious antibodies gradually damage multiple organ systems, falls within this scope. Since the 1950s, SLE 5 year survival rates from diagnosis have increased from ~50% to ~99% [1,2]. Therefore, there is a high demand for comprehensively identifying SLE-associated symptoms that negatively affect quality of life and establishing appropriate guidelines for their mitigation.

Voice disorders, or dysphonias, are impairments in the ability to vocalize. Social interactions rely heavily on speech, and therefore dysphonias can dramatically hinder communication and reduce life quality. Consequences of voice disorders include increased risk of depression and anxiety disorders, increase in stress as well as reduced self-reported measures of health [3–5]. Interestingly, dysphonia can be associated with some chronic diseases, including cystic fibrosis and Parkinson’s disease, contributing to the burden on patient wellbeing [6–8].

We hypothesized that SLE could lead to dysphonia due to progressive tissue damage. There have been reports of laryngeal and upper airway disorders in SLE patients [9–12], but a systematic relationship between SLE and dysphonia has not been investigated. We tested this hypothesis by measuring objective and subjective vocal parameters in SLE patients compared to matched controls, as well as assessing self-reported vocal deficits. We found that SLE patients have significant deficits in vocal quality, in both objective and subjective parameters, that correlate with measures of tissue damage.

## Methods

Selected subjects were females between 17 and 56 years of age diagnosed with SLE (n = 36) and a group of age and sex matched controls (n = 32) with no previous diagnosis of dysphonia or self-reported complaints in vocal quality or performance. All subjects had no gross abnormalities of the vocal folds, which was confirmed by laryngoscopic examination, and never received voice therapy. None of the subjects were smokers or heavy drinkers. SLE patients were receiving standard care from the Ophir Loyola Hospital and the Bettina Ferro de Sousa University Hospital, both located in Belém, Brazil. Attending physicians provided each patient’s current dosage of prednisone, time of diagnosis, including their SLICC/ACR (Systemic Lupus International Collaborating Clinics / American College of Rheumatology) damage index [13,14], assessed one week prior to vocal recordings. All subjects (or legal guardians in the case of minors) signed an informed consent form. This study was conducted according to the principles expressed in the Declaration of Helsinki and was approved by the ethics committee of the Ophir Loyola Hospital (CAAE:15241913.4.0000.0018).

Vocal recordings and analyses were performed as described in a previous study from our group [6]. In brief, subjects were requested to produce a sustained /a/ vowel phonation in their usual intensity for a maximum voicing time, which was recorded using a hand-held microphone (PG42-LC, Shure) positioned six centimeters from the subject’s mouth at a 45° angle. The initial second of the recording was always excluded from the analysis and parameters were measured over a three second time window following this period. Signals were recorded with a sampling rate of 44.1 kHz and analyzed with the Praat 6.0.13 software.

Five objective parameters of voice quality were measured: fundamental frequency (F_0_), intensity, jitter (index of F_0_ variability), shimmer (index of intensity variability) and the harmonics to noise ratio (HNR; index of glottal turbulence noise and hoarseness). These measurements are considered direct physical measures of vocal quality: low values of vocal intensity and HNR, high values of jitter and shimmer and altered F_0_ are considered potential signs of dysphonia [6,15]. Qualitative vocal parameters were assessed using the GRBAS (Grade, Roughness, Breathiness, Asthenia, Strain) scale, an extensively validated instrument for perceptual voice quality evaluation [16–18]. This method evaluates a subject’s general grade of dysphonia (G) by means of four subjective parameters: roughness (R), breathiness (B), asthenia (A), and strain (S). Each variable is scored on a scale from zero (normal) to three (severe abnormality). The evaluator was blind to subject identification. SLE patients also filled a questionnaire where they reported perceived deficits in their voice quality and capacity (S1 Table).

Most analyzed variables were not normally distributed (Shapiro-Wilk normality test; *P* > 0.05), and therefore all data are represented as scatter plots with median values added when necessary (individual values of each vocal parameter for the SLE and control groups are listed in S2 and S3 Tables, respectively; details on age, medication and time since diagnosis are listed in S4 Table). All comparisons of vocal parameters between control and SLE patients were evaluated with the non-parametric Mann-Whitney test. Potential correlations between variables were tested with linear regression analyses. For correlating SLICC/ACR damage scores with vocal variables, all individual symptom scores were summed (summed damage score), as well as the scores for each major organ system symptom category [13] which affected at least six patients (namely, renal, pulmonary, cardiovascular, peripheral vascular, gastrointestinal, musculoskeletal and skin; a detailed account of the SLICC/ACR damage scores for each patient is listed in S5 Table). Statistical significance level was set at *P* < 0.05 for all analyses.

## Results

**Fig 1:**
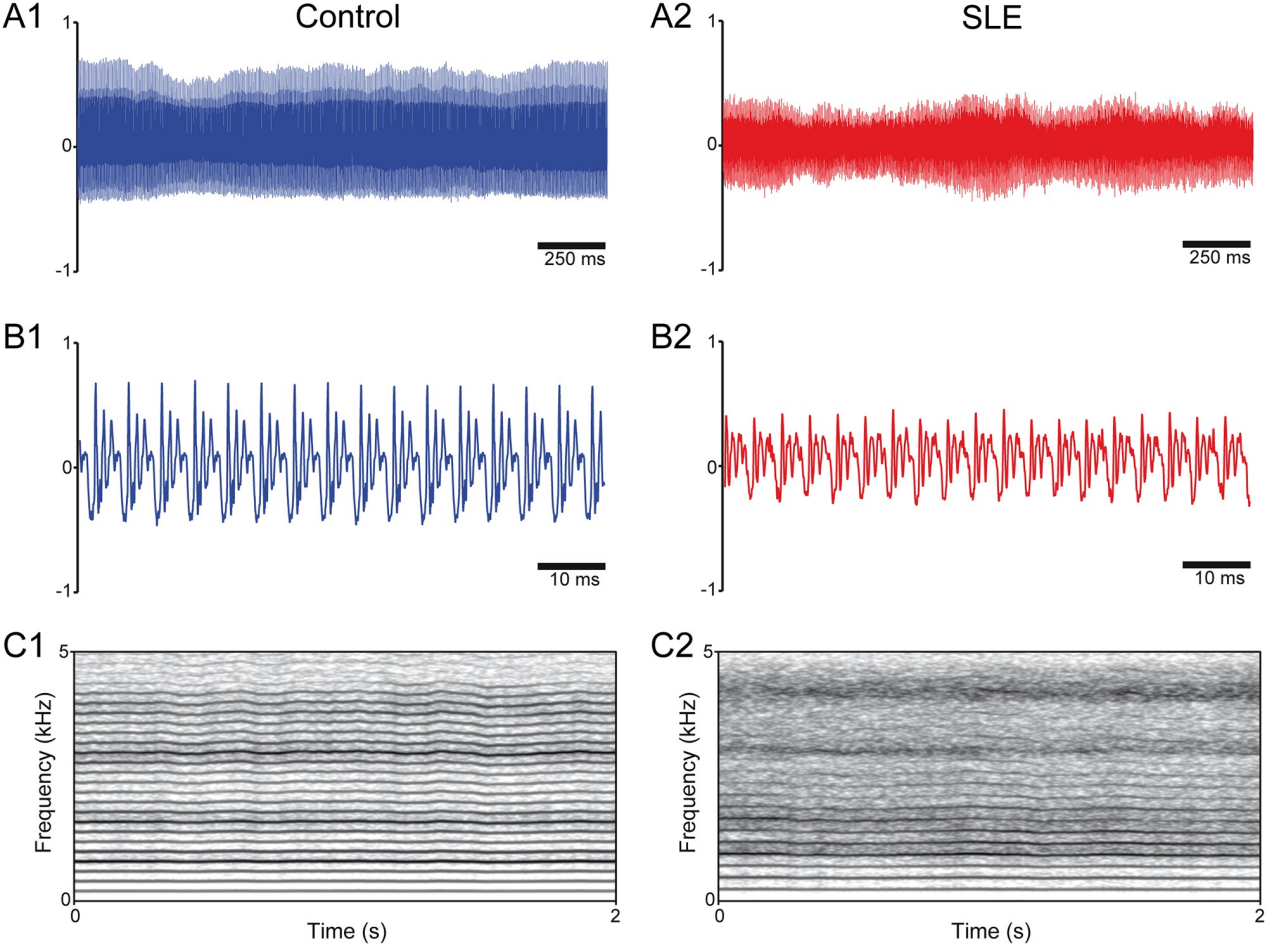
Representative voice recordings and spectrograms of SLE patients and healthy controls. **A:** Sound recordings of /a/ vowel phonations from a control subject (A1) and a patient with SLE (A2); note the high variability of the signal from the SLE patient in relation to the control. **B:** Zoomed in views of the recordings presented in A1 and A2; there is a noticeable reduction in the amplitude range in the SLE patient voical signal in relation to the representative control recording. **C:** Spectrogram (frequency domain) representations of the recordings presented in A1 and A2; note the high level of background noise and low formant segregation in the SLE patient compared to the healthy control.

**Fig 2:**
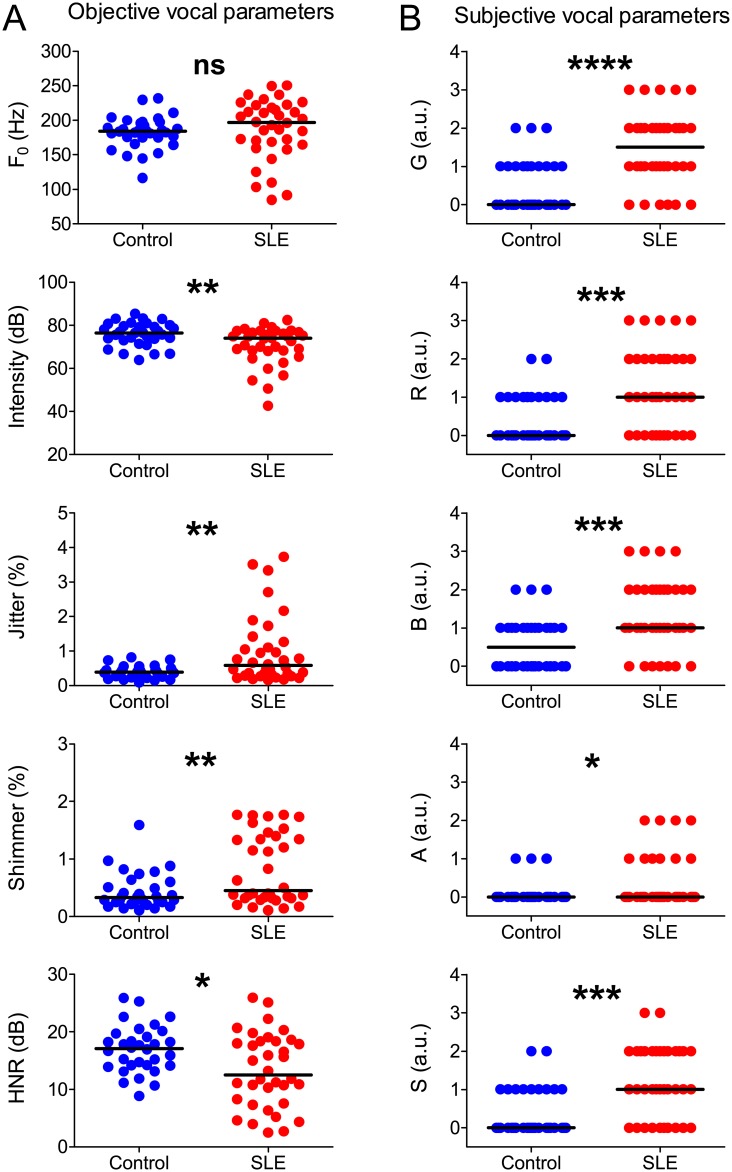
Objective and subjective (GRBAS) vocal parameters of control subjects (n = 32) and SLE patients (n = 36). Horizontal lines represent the population median. Each graph shows values for healthy controls and SLE patients of **A:** F_0_; **B:** vocal intensity; **C:** jitter (main formant frequency variability); **D:** shimmer (intensity variability); **E:** HNR; **F:** G (general grade of dysphonia); **G:** R (roughness); **H:** B (breathiness); **I:** A (asthenia); **J:** S (strain); * = *P* < 0.05; ** = *P* < 0.001. *** = *P* < 0.0001.

Vocal recordings and spectrograms of representative individuals are shown in Fig 1. Recordings from control subjects (Fig 1A1 and 1B1) showed standard features of euphonic voice, including low waveform variability and high periodic amplitude modulation, as well as spectrograms (Fig 1C1) with well-defined formant frequencies and low noise [6,19]. In contrast, vocal signals from SLE subjects showed reduced amplitude and high variability (Fig 2A2 and 2B2), in addition to spectrograms with high noise and dampened formant frequencies (Fig 1C2).

Analysis of objective vocal parameters (Fig 2A) revealed that, in relation to controls, SLE patients had lower vocal intensity and HNR, as well as increased jitter and shimmer. No differences were observed in F_0_. SLE patients also had higher scores of all components of the GRBAS scale (Fig 2B). Congruently, the majority (29/36) of SLE patients reported at least one perceived vocal deficit, with the most prevalent deficits being vocal fatigue (19/36) and hoarseness (17/36).

**Fig 3:**
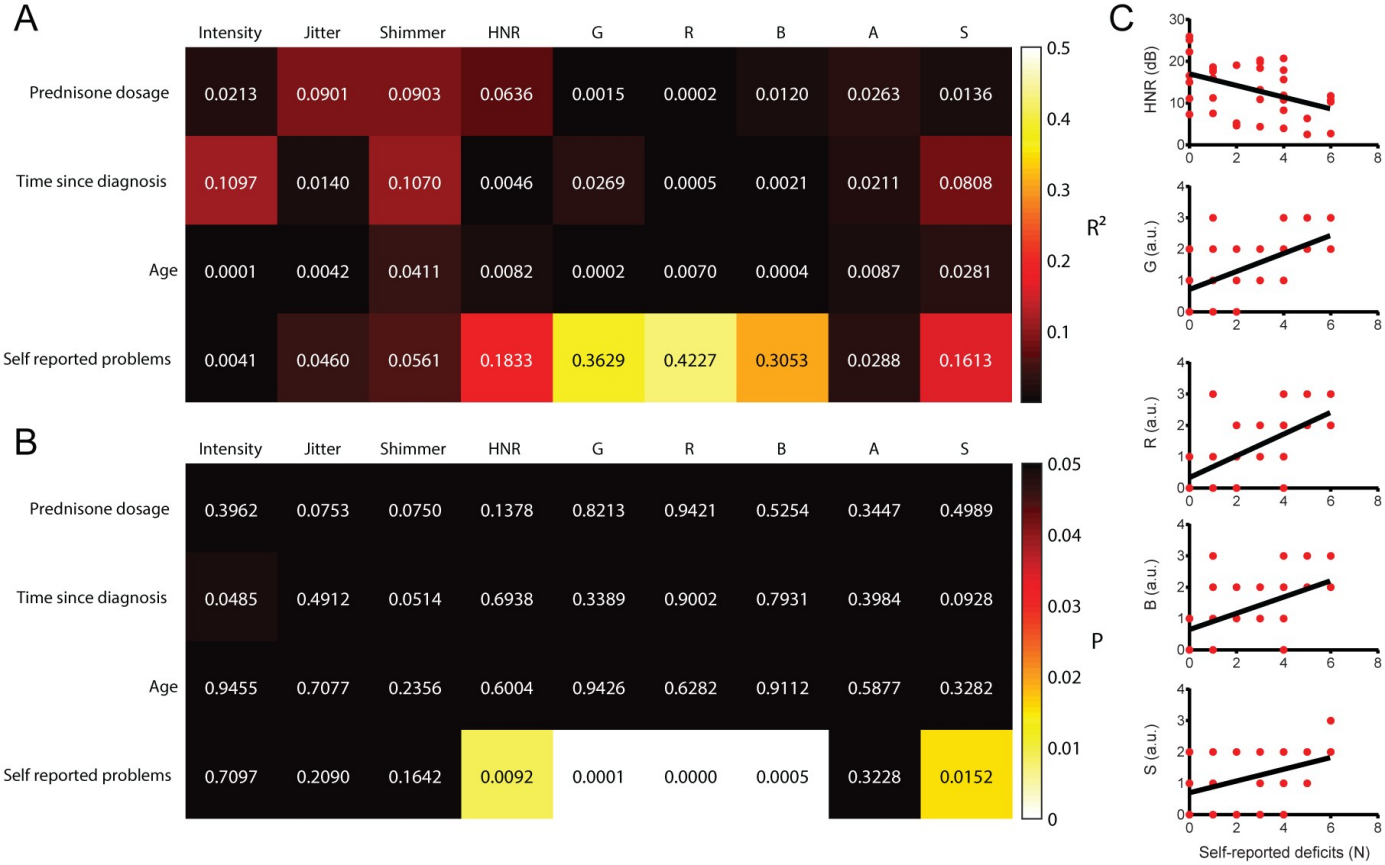
Linear regressions between objective and subjective vocal parameters with potential determinants of dysphonia and self-reported vocal deficits. **A:** Matrix representing the R^2^ values of linear regressions between selected variables. Note the high correlations (R^2^ > 0.15) between HNR, G, B, R and S with the number of self-reported vocal deficits. **B:** Matrix representing the *P* values of linear regressions between selected variables. Note the significant correlations (*P* < 0.05) between HNR, G, B, R and S with the number of self-reported vocal deficits. **C**: Scatter-plot representations of the significant correlations identified in the correlation matrices in A and B.

We tested whether objective vocal parameters and GRBAS scores correlated to features of patients’ medical history and self-reported vocal deficits (Fig 3). Scores of G, R, B and S, as well as HNR values, correlated with the total number of self-reported vocal deficits. Interestingly, time since diagnosis, prednisone dosage or age were not correlated to any change in objective or subjective vocal parameters.

**Fig 4:**
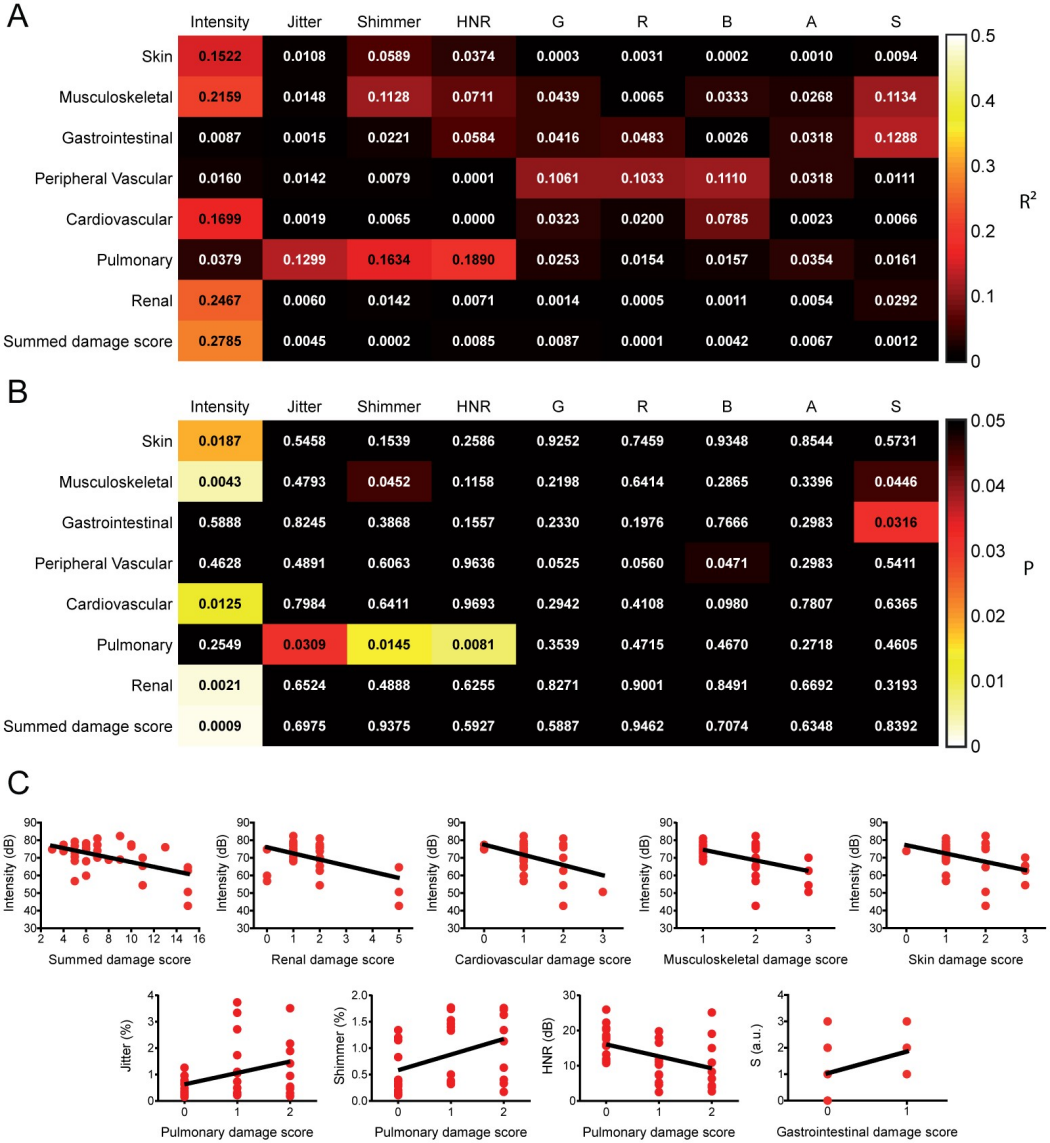
Linear regressions between objective and subjective vocal parameters with tissue damage as measured by the SLICC/ACR damage index. **A:** Matrix representing the R^2^ values of linear regressions between selected variables. Note the high correlations (R^2^ > 0.15) between intensity with summed damage scores and renal, cardiovascular, musculoskeletal and skin scores, as well as between pulmonary damage scores with jitter, shimmer and HNR. **B:** Matrix representing the *P* values of linear regressions between selected variables. Note the significant correlations (*P* < 0.05) between intensity with summed damage scores and renal, cardiovascular, musculoskeletal and skin scores, as well as between pulmonary damage scores with jitter, shimmer and HNR. **C**: Scatter-plot representations of selected significant correlations identified in the correlation matrices in A and B.

The summed SLICC/ACR damage index score showed a high anti-correlation with vocal intensity (R^2^ > 0.27, *P* = 0.0009; Fig 4), but not with any other vocal parameter. When we partitioned this summed score into different symptoms categories, we observed that renal, cardiovascular, musculoskeletal and skin symptoms were, by themselves, also anti-correlated with vocal intensity, albeit with lower determination coefficients and statistical significance (Fig 4A and 4B). Surprisingly, pulmonary damage scores were positively correlated with shimmer and jitter values, and negatively correlated with HNR. Likewise, gastrointestinal scores were positively correlated with S values. Additional, but very weak (R^2^ < 0.15, *P* ≈ 0.045), correlations were observed between musculoskeletal scores with shimmer and S, and peripheral vascular scores and B.

## Discussion

Our results show that a large fraction of SLE patients have dysphonic symptoms. SLE impacted vocal intensity, jitter, shimmer and HNR and all measures of the GRBAS scale. The vast majority of SLE patients also reported perceived deficits in vocal capacity. Most self-reported deficits—vocal fatigue and hoarseness—are consistent with changes in intensity and HNR, respectively, and are congruent with the altered scores in the GRBAS scale [6,20].

Affected vocal parameters did not correlate with age in either SLE patients or controls, indicating that age is not a determining factor in SLE-related dysphonia. Steroidal therapy has been reported to generate vocal impairments [21]; however, prednisone dosage did not correlate with vocal parameters, suggesting that steroid therapy is unlikely to be a major factor in SLE-related dysphonia.

HNR was negatively correlated, and G, R, B and S were positively correlated with the number of self-reported vocal deficits. This is an important confirmation that the reduction in objective and perceptual vocal quality observed in SLE patients is perceived by them, leading to subjective dissatisfaction. Furthermore, it is a strong indication that SLE-related dysphonic symptoms could potentially have a direct psychological effect on patients [22].

Summed SLICC/ACR damage scores were highly anti-correlated with vocal intensity, suggesting that the severity of SLE-related dysphonia is associated with tissue damage resulting from the disease. Interestingly, this correlation with intensity was also observed with the scores for renal, cardiovascular, musculoskeletal and skin symptoms. This result is perplexing, as intuitively only musculoskeletal symptoms could have a direct effect on patient vocal capacity, as damage to laryngeal and respiratory muscles can potentially lead to dysphonia [23,24]. Additionally, the summed damage score was a much better predictor of intensity changes than any individual symptom group. We hypothesize that, while muscular damage may be a partial cause of SLE-related intensity reductions, it is more likely that that the reduction in intensity is mostly due to a specific form of tissue damage that is not accounted for in the calculation of the SLICC/ACR damage index score. This could perhaps be direct tissue damage to the vocal cords or upper airways. If this is the case, the summed score, and some individual scores, would be correlated with intensity because they covary with the form of damage that is the direct cause of intensity reductions. Most interestingly, only pulmonary damage scores correlated with changes in the spectral properties of voice, namely jitter, shimmer and HNR, but not with intensity. Some pulmonary disorders, such as chronic obstructive pulmonary disease, have been linked to increases in jitter and shimmer and reductions in vocal quality [25], which would implicate that pulmonary damage could be a causal contributor to SLE-related changes in voice quality. Likewise, gastrointestinal scores were positively correlated with S values, albeit weakly. As some gastric disorders, most notably gastroesophageal reflux, have also been implicated in the origin of dysphonias [26,27], it is possible that gastrointestinal symptoms may contribute to SLE-related dysphonia.

Notably, no quantification of tissue damage correlated with changes in subjective vocal parameters, despite the high correlation between GRBAS scores and self-reported vocal complaints. We believe this reinforces our hypothesis that there are one or more causal origins of the identified SLE-related dysphonia that were undetectable by our methods of analysis.

Taken together, the results of our damage score-vocal parameter correlations indicate that SLE-related dysphonia is likely multifactorial—involving most likely pulmonary, gastrointestinal, musculoskeletal symptoms and other forms of injuries—and that specific individual combinations of tissue damage may lead to particular symptomatologies. However, the limitations of our study only allow us to speculate on this matter. Future studies, which must include a thorough examination of patient’s vocal apparatus and larger sample sizes, will be needed to confirm or reject this hypothesis.

Our results open the possibility that a considerable fraction of SLE patients may benefit from voice therapy [28]. This is especially important given that SLE patients have a high risk of developing depression and anxiety disorders [29,30], which can be exacerbated by deficits in socialization resulting from voice disorders [3–5]. Importantly, voice therapy has been shown to be the optimal strategy for mitigating dysphonic symptoms in other chronic diseases, including vocal deficits due to upper airway tissue damage [8,31,26]. Given our expectation that the etiology of SLE-related dysphonia is multifactorial and may vary widely between patients, it would be paramount to conduct individualized vocal evaluations before establishing the optimal therapeutic strategy. We tentatively conclude that including vocal evaluations and voice therapy in the guidelines for anamnesis and long-term care of SLE may potentially improve patient life quality and reduce disease burden.

## Supporting information

S1 Table Perceived vocal deficit questionnaire results, filled in by SLE patients in out cohort. Note that most (29/36) SLE patients reported at least one perceived vocal deficit, with the most prevalent deficits being vocal fatigue (19/36) and hoarseness (17/36).
(DOCX)

S2 Table Individual values of all measured objective and subjective vocal parameters for each SLE patient, as well as the means and medians for the group. (DOCX)

S3 Table Individual values of all measured objective and subjective vocal parameters for each subject of the control group, as well as the means and medians for the group. (DOCX)

S4 Table Individual values of daily prescribed prednisone doses, time since diagnosis and age for each SLE-patient, as well as the means and medians for the group. (DOCX)

S5 Table Individual values of all SLICC/ACR damage scores for each symptom and for each SLE patient. (XLSX)
