# Supplementary material for: Voice disorder in systemic lupus erythematosus
Source: PLoS One. 2017 Apr 17;12(4):e0175893. doi: 10.1371/journal.pone.0175893 (PMC5393869; doi:10.1371/journal.pone.0175893)
Supplement: S1 Table — Note that most (29/36) SLE patients reported at least one perceived vocal deficit, with the most prevalent deficits being vocal fatigue (19/36) and hoarseness (17/36). (DOCX) [file pone.0175893.s001.docx]

| Patient number | Vocal fatigue | Hoarseness | Voice became deeper | Voice became higher | Voice breaks/ sonority changes | Hoarseness after extended periods of talking | Voice becomes deeper after extended talking | Total number of perceived vocal deficits |
| --- | --- | --- | --- | --- | --- | --- | --- | --- |
| 1 | X | X | X |  | X |  |  | **4** |
| 2 |  |  |  |  |  |  |  | **0** |
| 3 |  |  |  |  |  |  |  | **0** |
| 4 | X | X |  | X |  |  |  | **3** |
| 5 |  | X | X |  |  |  |  | **2** |
| 6 |  |  | X |  | X |  | X | **3** |
| 7 |  |  |  |  |  |  |  | **0** |
| 8 |  | X | X |  | X |  | X | **4** |
| 9 | X | X | X |  | X | X | X | **6** |
| 10 | X |  | X |  | X |  | X | **4** |
| 11 |  | X |  |  |  |  |  | **1** |
| 12 | X |  |  | X |  | X | X | **4** |
| 13 |  |  |  |  |  |  |  | **1** |
| 14 | X |  |  | X | X |  |  | **3** |
| 15 |  | X |  |  |  |  |  | **1** |
| 16 | X | X |  |  |  |  |  | **2** |
| 17 |  | X |  | X |  | X |  | **3** |
| 18 | X | X | X |  | X | X |  | **5** |
| 19 |  | X | X |  |  |  |  | **2** |
| 20 | X | X | X |  |  | X | X | **5** |
| 21 |  |  |  |  |  |  |  | **0** |
| 22 |  |  |  |  |  |  |  | **0** |
| 23 | X |  |  | X | X |  |  | **3** |
| 24 | X | X | X |  | X | X | X | **6** |
| 25 | X | X |  | X | X | X | X | **6** |
| 26 | X |  | X |  |  | X |  | **3** |
| 27 |  |  |  | X | X | X |  | **3** |
| 28 |  |  |  |  |  |  |  | **0** |
| 29 | X |  | X |  |  | X | X | **4** |
| 30 | X |  |  |  |  |  |  | **1** |
| 31 | X | X | X |  |  |  | X | **4** |
| 32 | X | X | X |  | X | X | X | **6** |
| 33 |  | X | X |  |  | X | X | **4** |
| 34 |  |  |  |  |  |  |  | **0** |
| 35 | X |  |  |  |  |  |  | **1** |
| 36 | X |  |  |  |  |  |  | **1** |
| Total | **19** | **17** | **15** | **7** | **12** | **12** | **12** |  |

**Table legend**

Supplemental Table 1. Perceived vocal deficit questionnaire results, filled in by SLE patients in out cohort. Note that most (29/36) SLE patients reported at least one perceived vocal deficit, with the most prevalent deficits being vocal fatigue (19/36) and hoarseness (17/36).
