# Supplementary material for: Voice disorder in systemic lupus erythematosus
Source: PLoS One. 2017 Apr 17;12(4):e0175893. doi: 10.1371/journal.pone.0175893 (PMC5393869; doi:10.1371/journal.pone.0175893)
Supplement: S2 Table — (DOCX) [file pone.0175893.s002.docx]

| Patient number | F0  (Hz) | Intensity  (dB) | Jitter  (%) | Shimmer  (%) | HNR  (dB) | G  (a.u.) | R  (a.u.) | B  (a.u.) | A  (a.u.) | S  (a.u.) |
| --- | --- | --- | --- | --- | --- | --- | --- | --- | --- | --- |
| 1 | 168.65 | 65.41 | 0.35 | 0.28 | 17.88 | 1 | 1 | 1 | 0 | 0 |
| 2 | 206.87 | 50.63 | 0.54 | 0.4 | 15.01 | 1 | 1 | 1 | 0 | 1 |
| 3 | 230.62 | 70.09 | 0.16 | 0.11 | 25.94 | 1 | 0 | 1 | 0 | 1 |
| 4 | 196.26 | 54.43 | 0.19 | 0.14 | 20.3 | 2 | 1 | 2 | 1 | 2 |
| 5 | 211.53 | 62.68 | 0.47 | 0.33 | 19.07 | 1 | 1 | 1 | 1 | 2 |
| 6 | 193.09 | 59.86 | 0.36 | 0.32 | 18.36 | 2 | 2 | 1 | 0 | 2 |
| 7 | 205.58 | 69.01 | 0.18 | 0.17 | 25.11 | 2 | 0 | 1 | 2 | 2 |
| 8 | 218.23 | 42.72 | 0.29 | 1.13 | 8.28 | 3 | 3 | 3 | 1 | 0 |
| 9 | 172.45 | 70.13 | 1.05 | 0.38 | 11.73 | 3 | 3 | 3 | 2 | 3 |
| 10 | 84.61 | 64.61 | 1.42 | 0.63 | 3.96 | 1 | 0 | 0 | 0 | 1 |
| 11 | 202 | 56.79 | 0.62 | 0.37 | 17.62 | 2 | 1 | 2 | 0 | 2 |
| 12 | 170.66 | 74.81 | 0.78 | 1.15 | 10.76 | 2 | 2 | 2 | 0 | 0 |
| 13 | 109.76 | 68.27 | 0.22 | 1.4 | 7.54 | 1 | 0 | 1 | 0 | 1 |
| 14 | 225.74 | 77.43 | 0.47 | 1.2 | 13.22 | 1 | 1 | 1 | 0 | 0 |
| 15 | 237.16 | 72.71 | 0.66 | 1.34 | 11.24 | 0 | 0 | 1 | 0 | 0 |
| 16 | 91.5 | 68.05 | 3.73 | 1.46 | 5.22 | 0 | 0 | 1 | 0 | 0 |
| 17 | 186.87 | 76.01 | 3.51 | 1.76 | 4.36 | 1 | 1 | 1 | 0 | 1 |
| 18 | 249.78 | 77.6 | 0.95 | 1.73 | 6.34 | 2 | 2 | 2 | 0 | 1 |
| 19 | 172.85 | 76.79 | 3.34 | 1.77 | 4.62 | 2 | 2 | 2 | 0 | 2 |
| 20 | 143.76 | 77.37 | 2.71 | 1.74 | 2.51 | 3 | 3 | 3 | 2 | 2 |
| 21 | 221.89 | 82.4 | 1.73 | 1.33 | 11.08 | 0 | 0 | 0 | 0 | 1 |
| 22 | 226.39 | 68.27 | 1.1 | 1.77 | 7.31 | 0 | 0 | 0 | 0 | 0 |
| 23 | 236.96 | 76.52 | 1.89 | 1.34 | 10.89 | 2 | 2 | 2 | 0 | 1 |
| 24 | 103.3 | 74.1 | 0.73 | 1.53 | 10.29 | 2 | 2 | 2 | 0 | 2 |
| 25 | 125.01 | 68.94 | 2.17 | 1.63 | 2.72 | 2 | 2 | 2 | 0 | 2 |
| 26 | 250.44 | 74.8 | 0.35 | 0.37 | 18.36 | 1 | 1 | 1 | 0 | 1 |
| 27 | 159.74 | 79.19 | 0.27 | 0.32 | 19.8 | 2 | 2 | 1 | 2 | 2 |
| 28 | 185.53 | 75.15 | 0.49 | 0.35 | 16.54 | 1 | 1 | 0 | 1 | 1 |
| 29 | 210.45 | 75.14 | 0.76 | 0.38 | 15.65 | 1 | 0 | 0 | 1 | 1 |
| 30 | 215.17 | 70.7 | 0.38 | 0.29 | 18.6 | 0 | 0 | 0 | 0 | 0 |
| 31 | 164.62 | 78.29 | 0.22 | 0.16 | 20.68 | 2 | 2 | 2 | 1 | 2 |
| 32 | 222.59 | 74.91 | 1.26 | 0.83 | 10.73 | 3 | 3 | 2 | 0 | 3 |
| 33 | 197.6 | 75.61 | 0.96 | 0.4 | 11.86 | 2 | 2 | 2 | 0 | 2 |
| 34 | 212.08 | 73.85 | 0.27 | 0.2 | 22.25 | 0 | 0 | 0 | 0 | 0 |
| 35 | 184.35 | 77.43 | 0.23 | 0.32 | 15.93 | 1 | 1 | 1 | 0 | 0 |
| 36 | 157.63 | 81.02 | 0.31 | 0.5 | 17.98 | 3 | 3 | 3 | 0 | 2 |
| Mean | **187.55** | **70.60** | **0.98** | **0.82** | **13.33** | **1.47** | **1.25** | **1.33** | **0.39** | **1.19** |
| Median | **184.075** | **76.455** | **0.39** | **0.33** | **17.115** | **0** | **0** | **0.5** | **0** | **0** |

**Table legend**

Supplemental Table 2. Individual values of all measured objective and subjective vocal parameters for each SLE patient, as well as the means and medians for the group.
