# Supplementary material for: Voice disorder in systemic lupus erythematosus
Source: PLoS One. 2017 Apr 17;12(4):e0175893. doi: 10.1371/journal.pone.0175893 (PMC5393869; doi:10.1371/journal.pone.0175893)
Supplement: S3 Table — (DOCX) [file pone.0175893.s003.docx]

| Subject number | F0  (Hz) | Intensity  (dB) | Jitter  (%) | Shimmer  (%) | HNR  (dB) | G  (a.u.) | R  (a.u.) | B  (a.u.) | A  (a.u.) | S  (a.u.) |
| --- | --- | --- | --- | --- | --- | --- | --- | --- | --- | --- |
| 1 | 164.26 | 79.22 | 0.5 | 0.35 | 15.26 | 1 | 1 | 1 | 0 | 1 |
| 2 | 197.51 | 80.69 | 0.15 | 0.11 | 25.92 | 0 | 0 | 0 | 0 | 0 |
| 3 | 210.63 | 78.1 | 0.34 | 0.17 | 18.38 | 0 | 0 | 1 | 0 | 0 |
| 4 | 231.85 | 76.42 | 0.38 | 0.34 | 17.27 | 1 | 1 | 1 | 0 | 0 |
| 5 | 116.44 | 76.49 | 0.36 | 0.49 | 13.12 | 1 | 1 | 1 | 0 | 1 |
| 6 | 144.72 | 70.83 | 0.73 | 0.97 | 11.89 | 0 | 0 | 1 | 0 | 0 |
| 7 | 204.07 | 79.12 | 0.17 | 0.14 | 25.28 | 0 | 0 | 0 | 0 | 1 |
| 8 | 189.08 | 74.9 | 0.45 | 0.22 | 14.74 | 2 | 2 | 2 | 1 | 2 |
| 9 | 175.81 | 81.22 | 0.27 | 0.2 | 22.64 | 0 | 0 | 0 | 0 | 0 |
| 10 | 182.36 | 73.93 | 0.55 | 0.27 | 16.96 | 0 | 0 | 0 | 0 | 0 |
| 11 | 187.32 | 77.26 | 0.22 | 0.14 | 22.58 | 0 | 0 | 1 | 0 | 0 |
| 12 | 229.42 | 78.5 | 0.29 | 0.25 | 20.1 | 0 | 0 | 0 | 0 | 1 |
| 13 | 174.94 | 63.94 | 0.82 | 0.6 | 10.69 | 1 | 0 | 1 | 1 | 0 |
| 14 | 183.64 | 66.59 | 0.56 | 0.37 | 14.2 | 0 | 0 | 0 | 0 | 0 |
| 15 | 152.27 | 71.45 | 0.44 | 0.35 | 13.92 | 1 | 1 | 1 | 0 | 0 |
| 16 | 197.43 | 75.61 | 0.43 | 0.29 | 14.13 | 2 | 2 | 2 | 0 | 1 |
| 17 | 195.91 | 77.04 | 0.37 | 0.74 | 18.23 | 0 | 0 | 0 | 0 | 1 |
| 18 | 200.13 | 85.45 | 0.46 | 0.17 | 21.27 | 0 | 0 | 0 | 0 | 0 |
| 19 | 181.04 | 80.97 | 0.35 | 0.41 | 17.87 | 1 | 1 | 1 | 0 | 0 |
| 20 | 174.89 | 82.96 | 0.58 | 0.27 | 17.69 | 1 | 0 | 1 | 0 | 1 |
| 21 | 165.62 | 80.04 | 0.44 | 0.32 | 17.83 | 1 | 0 | 1 | 0 | 1 |
| 22 | 176.46 | 83.14 | 0.42 | 0.27 | 19.71 | 0 | 0 | 1 | 0 | 0 |
| 23 | 184.9 | 79.65 | 0.47 | 0.28 | 20.5 | 0 | 0 | 1 | 0 | 0 |
| 24 | 202.48 | 66.76 | 0.75 | 0.51 | 13.14 | 0 | 0 | 0 | 0 | 0 |
| 25 | 184.51 | 74.22 | 0.17 | 0.29 | 16.67 | 0 | 0 | 0 | 0 | 0 |
| 26 | 192.33 | 73.67 | 0.19 | 0.82 | 15.18 | 0 | 0 | 0 | 0 | 0 |
| 27 | 185.37 | 83.04 | 0.09 | 0.64 | 18.24 | 0 | 0 | 0 | 0 | 0 |
| 28 | 148.09 | 74.3 | 0.3 | 0.39 | 15.93 | 0 | 1 | 0 | 0 | 0 |
| 29 | 180.9 | 75.6 | 0.23 | 0.25 | 19.12 | 0 | 1 | 0 | 0 | 0 |
| 30 | 156.67 | 68.73 | 0.4 | 0.78 | 11.13 | 2 | 1 | 2 | 0 | 2 |
| 31 | 186.68 | 66.73 | 0.41 | 1.59 | 8.84 | 1 | 1 | 0 | 1 | 0 |
| 32 | 181.89 | 73.04 | 0.26 | 0.88 | 14.21 | 1 | 1 | 0 | 0 | 1 |
| Mean | **182.49** | **75.93** | **0.39** | **0.43** | **16.96** | **0.50** | **0.44** | **0.59** | **0.09** | **0.41** |
| Median | **196.93** | **73.975** | **0.58** | **0.45** | **12.54** | **1.5** | **1** | **1** | **0** | **1** |

**Table legend**

Supplemental Table 3. Individual values of all measured objective and subjective vocal parameters for each subject of the control group, as well as the means and medians for the group.
