# Supplementary material for: Voice disorder in systemic lupus erythematosus
Source: PLoS One. 2017 Apr 17;12(4):e0175893. doi: 10.1371/journal.pone.0175893 (PMC5393869; doi:10.1371/journal.pone.0175893)
Supplement: S4 Table — (DOCX) [file pone.0175893.s004.docx]

| Patient number | Prednisone dosage (mg/day) | Time since diagnosis (months) | Age (years) |
| --- | --- | --- | --- |
| 1 | 5 | 84 | 45 |
| 2 | 0 | 39 | 28 |
| 3 | 15 | 36 | 55 |
| 4 | 0 | 336 | 47 |
| 5 | 20 | 144 | 40 |
| 6 | 7.5 | 132 | 34 |
| 7 | 40 | 8 | 30 |
| 8 | 60 | 21 | 19 |
| 9 | 10 | 132 | 50 |
| 10 | 5 | 360 | 55 |
| 11 | 5 | 204 | 49 |
| 12 | 0 | 48 | 18 |
| 13 | 40 | 2 | 32 |
| 14 | 40 | 8 | 48 |
| 15 | 10 | 19 | 23 |
| 16 | 60 | 0.1 | 23 |
| 17 | 45 | 15 | 22 |
| 18 | 10 | 120 | 32 |
| 19 | 15 | 72 | 40 |
| 20 | 10 | 96 | 56 |
| 21 | 10 | 18 | 48 |
| 22 | 0 | 52 | 31 |
| 23 | 5 | 52 | 18 |
| 24 | 10 | 16 | 33 |
| 25 | 30 | 14 | 42 |
| 26 | 0 | 36 | 17 |
| 27 | 25 | 48 | 17 |
| 28 | 15 | 120 | 34 |
| 29 | 10 | 24 | 29 |
| 30 | 7.5 | 36 | 40 |
| 31 | 10 | 120 | 50 |
| 32 | 10 | 26 | 22 |
| 33 | 15 | 96 | 31 |
| 34 | 5 | 72 | 31 |
| 35 | 5 | 29 | 55 |
| 36 | 15 | 48 | 35 |
| Mean | **15.83** | **74.53** | **35.53** |
| Median | **10.00** | **48.00** | **33.50** |

**Table legend**

Supplemental Table 4. Individual values of daily prescribed prednisone doses, time since diagnosis and age for each SLE-patient, as well as the means and medians for the group.
